# Metabolomic Profiling of Serum Retinol in the Alpha-Tocopherol, Beta-Carotene Cancer Prevention (ATBC) Study

**DOI:** 10.1038/s41598-017-09698-w

**Published:** 2017-09-06

**Authors:** Jiaqi Huang, Orestis A. Panagiotou, Gabriella M. Anic, Alison M. Mondul, Linda M. Liao, Andriy Derkach, Rachael Stolzenberg-Solomon, Stephanie J. Weinstein, Demetrius Albanes

**Affiliations:** 1 Division of Cancer Epidemiology and Genetics, National Cancer Institute, National Institutes of Health, Bethesda, MD USA; 2 Department of Epidemiology, University of Michigan School of Public Health, Ann Arbor, MI USA

## Abstract

The role of retinol in the prevention of multifactorial chronic diseases remains uncertain, and there is sparse evidence regarding biological actions and pathways implicated in its effects on various outcomes. The aim is to investigate whether serum retinol in an un-supplemented state is associated with low molecular weight circulating metabolites. We performed a metabolomic analysis of 1,282 male smoker participants based on pre-supplementation fasting serum in the Alpha-Tocopherol, Beta-Carotene Cancer Prevention (ATBC) Study. We examined the association between 947 metabolites measured by ultra-high performance LC-MS/GC-MS and retinol concentration (from HPLC) using linear regression that estimated the difference in metabolite concentrations per unit difference in retinol concentration as standardized β-coefficients and standard errors (SE). We identified 63 metabolites associated with serum retinol below the Bonferroni-corrected P-value (p < 5.3 × 10^–5^). The strongest signals were for N-acetyltryptophan (β = 0.27; SE = 0.032; p = 9.8 × 10^−17^), myo-inositol (β = 0.23; SE = 0.032; p = 9.8 × 10^−13^), and 1-palmitoylglycerophosphoethanolamine (β = 0.22; SE = 0.032; p = 3.2 × 10^−12^). Several chemical class pathways were strongly associated with retinol, including amino acids (p = 1.6 × 10^−10^), lipids (p = 3.3 × 10^–7^), and cofactor/vitamin metabolites (3.3 × 10^−7^). The strongest sub-pathway association was for inositol metabolism (p = 2.0 × 10^–14^). Serum retinol concentration is associated with circulating metabolites in various metabolic pathways, particularly lipids, amino acids, and cofactors/vitamins. These interrelationships may have relevance to the biological actions of retinol, including its role in carcinogenesis.

## Introduction

The importance of vitamin A compounds for homeostasis and normal physiology is well-established^1^, and its key biological functions include critical roles in embryonic development and growth, cell differentiation, tissue remodeling, reproduction, integrity of the immune system, vision, maintenance of skin and membranes, and hematopoiesis^2–6^. Vitamin A deficiency can lead to xerophthalmia, blindness, infections, and even death, especially in low-income settings^7^. Retinol and vitamin A compounds including retinoic acid are also integral to lipid metabolism, insulin signaling, and energy balance^4–6^, and they exert pleiotropic effects by regulating or co-regulating the expression of over 500 genetic response elements after binding with its nuclear receptors retinoic acid receptor (RAR), retinoid X receptor (RXR), and peroxisome proliferator-activated receptor β/δ (PPAR β/δ), as well as heterodimerizing with the vitamin D and thyroid hormone receptors^8^. These biological actions are thought to be responsible for their experimental anti-carcinogenic effects^9–12^, although increased cell proliferation and decreased cell differentiation have also been observed^13^.

The role of vitamin A in the prevention of common chronic diseases is less clear, however^14, 15^. Early pre-clinical and population-based observational studies^10^ suggested protective effects of retinol on cancer^11, 16^ and cardiovascular disease^17–19^. By contrast, subsequent evidence from randomized trials and meta-analyses^20^ have not supported many of the observational findings^21^, instead showing increased risk for some outcomes such as cancer of the prostate^22, 23^ and lung^24^, cardiovascular disease^25^, and even overall mortality^24^ for individuals with high circulating retinol concentration or following supplementation with vitamin A or β-carotene^14, 23, 24, 26^. For example, a recent pooled analysis of 15 cohort studies that included more than 11,000 prostate cancer cases found 13% higher prostate cancer risk in the highest versus lowest category of serum retinol^23^. Such data have led the U.S. Preventive Services Task Force to question the public health benefits of supplementation with vitamin A in the absence of deficiency^14, 27^.

Despite evidence from molecular and laboratory studies that metabolic derivatives of retinol could promote carcinogenesis^28, 29^, the relevant biologic pathways are not understood. Elucidating the biological mechanisms underlying these vitamin A associations would have implications for any future prevention trials, selection of their target populations, and general population vitamin A supplementation^30^.

Here, we hypothesized that serum retinol in an un-supplemented state may be associated with other small, low-molecular metabolites in circulation, and conducted an agnostic metabolomic analysis to identify biologically relevant metabolites related to vitamin A status.

## Results

Characteristics at study entry for the 1,282 participants included in this analysis are shown in Table 1. The median serum concentration of retinol was 579 μg/L. The median number of cigarettes smoked per day was 20 (interquartile range, IQR, 14–25), and 21% of participants exercised 3 times per week or more. Serum retinol <0.7 µmol/L (or 200 µg/L) is an accepted definition of vitamin A deficiency in most age groups^31, 32^. In the present subset of 1,208 men, only one had a borderline-deficient serum retinol value (i.e., 192 µg/L).

**Table 1:** Pre-randomization characteristics of 1,282 Finnish male smokers in the ATBC Study

Characteristic^1^	
Age at randomization (years), median (IQR)	58 (54–63)
Height (cm)	173.5 (6.1)
Weight (kg)	79.0 (12.9)
BMI (kg/m^2^)	26.2 (3.8)
**Smoking, median (IQR)**	
cigarettes/day	20 (14–25)
duration (years)	38 (32–43)
Physical activity (3 or more times/week, %)	21
**Dietary intake, median (IQR)**	
total energy, (kcal/d)	2640 (2170–3128)
fruit (g/d)	109 (60–175)
vegetables (g/d)	99 (63–149)
red meat (g/d)	66 (50–89)
coffee (g/d)	560 (440–840)
alcohol, ethanol (g/d)	10 (2–24)
vitamin A (µg/d)	1627 (1128–2248)
**Intervention group, N (%)**	
placebo	327 (26)
beta-carotene only	342 (27)
alpha-tocopherol only	299 (23)
alpha-tocopherol and beta-carotene	314 (24)
Serum retinol (ug/l), median (IQR)	579 (511–659)
Serum total cholesterol (mmol/l), median (IQR)	6.22 (5.45–6.94)

^1^Data for continuous variables are shown as mean (standard deviation) unless otherwise specified.

IQR: interquartile range.

In multivariable linear regression models, 263 metabolites were associated with retinol concentrations at *p* < 0.05 (Supplemental Table 1), of which 63 (Table 2) were significant after Bonferroni correction (*p* < 5.3 × 10^−5^). All of them were positively associated with a standard deviation (SD) increase in retinol, except for the peptide metabolite adsgegdfxaegggvr (fibrinopeptide A) that showed an inverse association (β = −0.16). The strongest association with serum retinol was seen for N-acetyltryptophan (β = 0.27 per SD increase in retinol concentration; *p* = 9.9 × 10^−17^), followed by the lipids *myo-*inositol and 1-palmitoylglycerophosphoethanolamine, the amino acid 4-acetamidobutanoate, and the purine N6-carbamoylthreonyladenosine (β = 0.23, *p* = 9.8 × 10^−13^; β = 0.22, *p* = 3.2 × 10^−12^; β = 0.22, *p* = 8.9 × 10^−12^; β = 0.21, *p* = 2.9 × 10^−11^

**Table 2:** Metabolites associated with serum retinol concentration at 5.3 × 10^−5^ level of statistical significance.

Metabolite^1^	Estimate^2^	Standard error	*p* value	Coefficient of variation	Chemical Sub-Class
**Amino acid and amino acid derivatives**					
N-Acetyltryptophan	0.272	0.0321	9.89 × 10^−17^	0.15	Tryptophan metabolism
4-Acetamidobutanoate	0.216	0.0314	8.87 × 10^−12^	0.18	Guanidino and acetamido metabolism; polyamine metabolism
N-Acetyl-3-methylhistidine	0.217	0.0331	9.84 × 10^−11^	0.49	Histidine metabolism
Homocitrulline	0.201	0.0320	4.87 × 10^−10^	0.14	Urea cycle; arginine and proline metabolism
N-Acetylvaline	0.192	0.0314	1.32 × 10^−09^	0.15	Valine, leucine and isoleucine metabolism
N-Acetyl-1-methylhistidine	0.200	0.0339	4.65 × 10^−09^	0.10	Histidine metabolism
1-Methylhistidine	0.187	0.0322	8.41 × 10^−09^	0.13	Histidine metabolism
N6-Acetyllysine	0.179	0.0323	4.23 × 10^−08^	0.08	Lysine metabolism
Creatinine	0.172	0.0322	1.11 × 10^−07^	0.13	Creatine metabolism
N-Acetylthreonine	0.162	0.0319	4.51 × 10^−07^	0.11	Glycine, serine and threonine metabolism
Beta-hydroxyisovaleroylcarnitine	0.181	0.0363	8.24 × 10^−07^	0.14	Valine, leucine and isoleucine metabolism
Indolelactate	0.159	0.0321	8.99 × 10^−07^	0.09	Tryptophan metabolism
N-Delta-acetylornithine	0.157	0.0321	1.14 × 10^−06^	0.24	Urea cycle; arginine and proline metabolism
N2,N5-Diacetylornithine	0.204	0.0425	2.06 × 10^−06^	0.13	Urea cycle; arginine and proline metabolism
Urea	0.151	0.0323	3.47 × 10^−06^	0.12	Urea cycle; arginine and proline metabolism
3-Methoxytyramine sulfate	0.197	0.0432	6.58 × 10^−06^	0.23	Phenylalanine & tyrosine metabolism
Guanidinosuccinate	0.182	0.0414	1.32 × 10^−05^	0.38	Guanidino and acetamido metabolism
3-Methylhistidine	0.140	0.0327	1.97 × 10^−05^	0.39	Histidine metabolism
Isovalerylcarnitine	0.132	0.0310	2.35 × 10^−05^	0.12	Valine, leucine and isoleucine metabolism
Tiglyl carnitine	0.138	0.0326	2.56 × 10^−05^	0.10	Valine, leucine and isoleucine metabolism
**Amino acid and amino acid derivatives; xenobiotics**					
2-Hydroxyisobutyrate	0.205	0.0357	1.22 × 10^−08^	0.10	Valine, leucine and isoleucine metabolism; chemical
**Lipids**					
*Myo-*inositol	0.228	0.0316	9.82 × 10^−13^	0.17	Inositol metabolism
1-Palmitoylglycerophosphoethanolamine	0.223	0.0316	3.16 × 10^−12^	0.35	Lysolipid
4-Androsten-3beta,17beta-diol monosulfate	0.254	0.0397	3.39 × 10^−10^	0.07	Sterol/steroid
1-Palmitoleoylglycerophosphocholine	0.195	0.0312	5.74 × 10^−10^	0.57	Lysolipid
Propionylcarnitine	0.198	0.0316	6.20 × 10^−10^	0.23	Fatty acid metabolism (also BCAA metabolism)
2-Palmitoleoylglycerophosphocholine	0.199	0.0323	1.10 × 10^−9^	0.78	Lysolipid
4-Androsten-3beta,17beta-diol disulfate	0.180	0.0301	3.35 × 10^−9^	0.04	Sterol/steroid
5Alpha-androstan-3beta,17beta-diol disulfate	0.189	0.0311	1.60 × 10^−9^	0.07	Sterol/steroid
Stearoyl-arachidonoyl-glycerophosphoethanolamine	0.223	0.0409	7.74 × 10^−8^	0.12	Lysolipid
Etiocholanolone glucuronide	0.210	0.0420	7.91 × 10^−7^	0.12	Sterol/steroid
1-Linolenoylglycerophosphocholine (18:3n3)	0.175	0.0354	9.65 × 10^−7^	0.41	Lysolipid
4-Androsten-3beta,17beta-diol disulfate	0.157	0.0318	9.83 × 10^−7^	0.06	Sterol/steroid
Inositol 1-phosphate (I1P)	0.148	0.0302	1.06 × 10^−6^	0.24	Inositol metabolism
Palmitoyl-oleoyl-glycerophosphocholine	0.201	0.0412	1.31 × 10^−6^	0.09	Lysolipid
Cortisol	0.157	0.0324	1.43 × 10^−6^	0.07	Sterol/steroid
1-Oleoylglycerol (1-monoolein)	0.160	0.0338	2.51 × 10^−6^	0.34	Monoacylglycerol
Acetylcarnitine	0.147	0.0315	3.73 × 10^−6^	0.28	Carnitine metabolism
2-Palmitoylglycerophosphocholine	0.148	0.0321	4.26 × 10^−6^	0.59	Lysolipid
Malonylcarnitine	0.196	0.0424	4.57 × 10^−6^	0.30	Fatty acid synthesis
Carnitine	0.140	0.0318	1.20 × 10^−5^	0.10	Carnitine metabolism
Scyllo-inositol	0.262	0.0591	1.31 × 10^−5^	0.48	Inositol metabolism
1-Oleoylglycerophosphocholine	0.137	0.0315	1.47 × 10^−5^	0.43	Lysolipid
2-Myristoylglycerol (2-monomyristin)	0.156	0.0363	1.94 × 10^−5^	0.42	Monoacylglycerol
1-Oleoylglycerophosphoethanolamine	0.138	0.0322	2.13 × 10^−5^	0.40	Lysolipid
1-Palmitoylglycerophosphocholine	0.138	0.0322	2.17 × 10^−5^	0.28	Lysolipid
2-Palmitoylglycerol (2-monopalmitin)	0.148	0.0347	2.33 × 10^−5^	0.43	Monoacylglycerol
21-Hydroxypregnenolone disulfate	0.135	0.0321	2.94 × 10^−5^	0.06	Sterol/steroid
Palmitoyl-arachidonoyl-glycerophosphocholine	0.165	0.0395	3.42 × 10^−5^	0.19	Lysolipid
1-Linolenoylglycerol	0.138	0.0332	3.72 × 10^−5^	0.20	
1-Stearoylglycerophosphoethanolamine	0.131	0.0321	4.64 × 10^−5^	0.42	Lysolipid
**Nucleotides**					
N6-Carbamoylthreonyladenosine	0.212	0.0315	2.91 × 10^−11^	0.09	Purine metabolism, guanine containing; purine metabolism, adenine containing
Urate	0.205	0.0308	4.14 × 10^−11^	0.07	Purine metabolism, urate metabolism; purine metabolism, (Hypo)xanthine/inosine containing
Pseudouridine	0.191	0.0318	2.81 × 10^−9^	0.1	Pyrimidine metabolism, uracil containing
N2,N2-Dimethylguanosine	0.134	0.0317	2.76 × 10^−5^	0.2	Purine metabolism, guanine containing
**Carbohydrates**					
Erythronate	0.198	0.0313	4.01 × 10^−10^	0.14	Aminosugar metabolism
Arabitol	0.188	0.0308	1.39 × 10^−9^	0.14	Pentose metabolism
**Xenobiotics**					
Erythritol	0.192	0.0313	1.10 × 10^−9^	0.14	Sugar, sugar substitute, starch; food component/plant
O-Sulfo-L-tyrosine	0.217	0.0358	2.13 × 10^−9^	0.08	Chemical
Ethyl glucuronide	0.164	0.0319	3.00 × 10^−7^	0.06	Detoxification and metabolism; chemical
3-Hydroxycotinine glucuronide	0.143	0.0302	2.34 × 10^−6^	0.08	Tobacco metabolite
**Peptides**					
Adsgegdfxaegggvr	−0.157	0.0353	9.38 × 10^−6^	0.05	Fibrinogen cleavage peptide
**Energy**					
Succinylcarnitine	0.134	0.0315	2.37 × 10^−5^	0.38	Krebs cycle / TCA cycle

^1^All metabolites had detectable values in > 80% of study population with the exception of N-acetyl-3-methylhistidine, N-acetyl-1-methylhistidine, beta-hydroxyisovaleroylcarnitine, N2,N5-diacetylornithine, 3-methoxytyramine sulfate, guanidinosuccinate, 4-androsten-3beta,17beta-diol monosulfate, 2-palmitoleoylglycerophosphocholine, stearoyl-arachidonoyl-glycerophosphoethanolamine, etiocholanolone glucuronide, 1-linolenoylglycerophosphocholine (18:3n3), palmitoyl-oleoyl-glycerophosphocholine, malonylcarnitine, scyllo-inositol, 2-myristoylglycerol (2-monomyristin), palmitoyl-arachidonoyl-glycerophosphocholine, 1-linolenoylglycerol and O-sulfo-L-tyrosine (71%, 77%, 68%, 46%, 45%, 45%, 47%, 78%, 47%, 47%, 68%, 47%, 47%, 39%, 66%, 47%, 79% and 68%, respectively).

^2^Estimate is the standardized beta-coefficient

, respectively). The findings were not altered by adjustment for serum creatinine. Also, results were the same after model adjustment for storage time (from blood collection to metabolomics measurement) (data not shown).

Of the metabolites that remained statistically significant after Bonferroni correction, the majority were lipids (n = 30 of 264 lipids) and amino acids (n = 20 of 157 amino acids). The remaining were nucleotides (n = 4 of 31), xenobiotics (n = 4 of 124), carbohydrates (n = 2 of 26), energy metabolites (n = 1 of 9), and peptides (n = 1 of 91). The metabolite chemical classes of amino acids, lipids, and cofactors/vitamins had strong positive associations with serum retinol (*p* = 1.6 × 10^−10^, 3.3 × 10^−7^ and 3.3 × 10^−7^, respectively; Table 3, Supplemental Table 2). In addition, 23 out of 59 metabolite chemical sub-classes exceeded the Bonferroni threshold (*P* < 8.5 × 10^−4^), with the top signals being for inositol, creatine, valine/leucine/isoleucine, and purine/urate pathways (*p* = 2.0 × 10^−14^, 1.4 × 10^−12^, 2.8 × 10^−11^, and 2.4 × 10^−10^, respectively; Table 4, Supplemental Table 3).

**Table 3:** Gene set analysis for chemical class of metabolites for serum retinol

Chemical Class	No. of contributing metabolites	No. of contributing metabolites after Bonferroni correction	*p* value
Amino acids	157	20	1.57 × 10^−10^
Lipids	264	30	3.28 × 10^−7^
Cofactors and vitamins	28	0	3.32 × 10^−7^
Energy	9	1	1.82 × 10^−5^
Peptides	91	1	8.58 × 10^−5^
Carbohydrates	26	2	9.22 × 10^−5^
Nucleotides	31	4	0.00011
Xenobiotics	124	4	0.04470

**Table 4:** Gene set analysis for chemical sub-class of metabolites for serum retinol at 8.5 × 10^−4^ level of statistical significance.

Sub-Pathway	No. of contributing metabolites	*p* value
Inositol metabolism	4	1.98 × 10^−14^
Creatine metabolism	3	1.41 × 10^−12^
Valine, leucine and isoleucine metabolism	27	2.75 × 10^−11^
Purine metabolism, urate metabolism; purine metabolism, (Hypo)xanthine/inosine containing	3	2.43 × 10^−10^
Lysolipid	62	1.02 × 10^−8^
Urea cycle; arginine and proline metabolism	14	1.76 × 10^−8^
Monoacylglycerol	13	2.24 × 10^−8^
Histidine metabolism	12	3.63 × 10^−8^
Tryptophan metabolism	18	1.60 × 10^−7^
Sterol/steroid	46	1.76 × 10^−7^
Lysine metabolism	9	1.99 × 10^−6^
Aminosugar metabolism	2	7.38 × 10^−6^
Fatty acid metabolism (also BCAA metabolism)	5	9.86 × 10^−6^
Polyamine metabolism	3	1.52 × 10^−5^
Fibrinogen cleavage peptide	2	3.79 × 10^−5^
Pentose metabolism	10	6.39 × 10^−5^
Ascorbate and aldarate metabolism	3	0.000126
Alanine and aspartate metabolism; pyrimidine metabolism, uracil containing	3	0.000238
Glutamate metabolism	6	0.000250
Pyrimidine metabolism, uracil containing	6	0.000295
Cysteine, methionine, SAM, taurine metabolism	15	0.000558
Glutathione metabolism	2	0.000630
Essential fatty acid; polyunsaturated fatty acid (N3 and N6)	7	0.000681

## Discussion

This large-scale agnostic investigation of nearly 1,300 men identified 63 metabolites in several chemical classes associated with serum retinol concentrations after stringent correction for 947 retinol-metabolite comparisons. Amino acids and lipids were most highly represented among the top metabolites, with N-acetyltrytophan and *myo-*inositol (followed by the lipid 1-palmitoylglycerophosphoethanolamine) ranked first within these two chemical classes, respectively. In addition, N6-carbamoylthreonyladenosine, erythronate, erythritol, adsgegdfxaegggvr, and succinylcarnitine were represented as the top metabolites within the nucleotide, carbohydrate, xenobiotic, peptide, and energy metabolite classes.

N-acetyltryptophan prevents oxidative degradation of proteins^33^, and the tryptophan/kynurenine pathway influences the regulation of inflammation, oxidative stress and immune activation^34, 35^. The relevance of several other N-acetyl amino acids among the top metabolites (e.g., N-acetyl-3-methylhistidine, -valine, −1-methylhistidine, -lysine, and -threonine) is unclear, but may indicate perturbations in acetylation activity and influence on cell growth through histone-chromatin function and gene regulation^36–38^.

Retinol plays an important role in lipid metabolism through activation of RAR and PPARβ/δ signaling and gene expression enhancing lipid accumulation through control of adipocyte differentiation, fatty acid oxidation, and lipolysis^39–43^. *Myo*-inositol ranked first among lipid metabolites associated with retinol, which along with its phosphorylated derivatives including inositol 1-phosphate (I1P), may be related to mediation of retinoic acid signaling and cellular functions including adhesion, growth, vesicular trafficking, and cell survival^44, 45^, and is involved in regulation of enzyme activity and hormone secretion^46^. Through its role in phosphatidylinositol-4-phosphate (IP_4_) and phosphatidylinositol-4,5-bisphosphate (IP_4, 5_) biosynthesis, *myo-*inositol and I1P are plasma membrane mediators of protein phosphorylation and second messenger signaling^47^.

Several lysolipids and androgen metabolites were associated with serum retinol. The former glycerophospho-fatty acids are also important plasma membrane cell-signaling molecules^48, 49^ that influence cell differentiation, growth, proliferation, and invasion^50–55^. The four serum androstenediol (adiol) sulfate metabolites positively related to serum retinol could serve as androgenic mediators and confer the vitamin A-prostate cancer association^56^.

The present analysis also shows retinol is correlated with histidine pathway metabolites, including N-acetyl-3-methylhistidine, N-acetyl-1-methylhistidine, 1-methylhistidine and 3-methylhistidine. Experimental data indicate that biosynthesis of histidine metabolites may be mediated through the 5-phosphoribosyl-1-pyrophosphate signaling pathway^57^. 1- and 3-Methylhistidine are abundant in actin, formed by post-translational methylation^58, 59^, and are considered markers of myofibrillar protein degradation^60^. Excessive vitamin A intake may stimulate 3-methylhistidine excretion both *in vivo* and *in vitro*, suggesting accelerated myofibrillar protein breakdown and protein turnover^61^. Aside from myofibrillar protein degradation, aberrations in histidine metabolites may also be indicative of tissue repair, tissue damage, alterations in the inflammatory signals and oxidative stress^62–65^.

This cross-sectional analysis was conducted in order to both contribute to elucidation of the molecular basis of retinol’s diverse biological activities and provide mechanistic clues related to how higher vitamin A status might adversely impact prostate (and other) cancer risk. In this regard, the present findings regarding inositol and lysolipid metabolites are of interest in that they (e.g., I1P) were associated with aggressive prostate cancer in our recent serum metabolomic analysis^66^. In addition, findings from our group based on size and extent of primary prostate cancer cases demonstrated a positive association with histidine metabolites including N-acetyl-3-methylhistidine, and 1- and 3-methylhistidine in T2 prostate cancers^67^. Some of the sex steroids related to serum retinol here were associated with non-aggressive prostate cancer the Prostate, Lung, Colorectal, and Ovarian Cancer Screening Trial^68^.

Fibrinopeptide A is the only metabolite we found to be inversely associated with serum retinol in this study. Fibrinopeptide A is produced from fibrinogen by thrombin during blood coagulation, and it is elevated in several malignancies, with persistent coagulation activation, thrombosis and disseminated intravascular coagulation being common complications^69–72^. Given that some cancers have been related to vitamin A status, the inverse fibrinopeptide A-retinol association observed here should be examined in other studies.

To our knowledge, this is the first metabolomic profiling analysis of vitamin A status. Strengths of the analysis include its relatively large sample size, assaying of serum collected after an overnight fast, and high laboratory quality control. Some limitations should also be acknowledged, including that our study was cross-sectional, which limits our ability to make causal inferences about the association between metabolites and retinol levels. In addition, the Alpha-Tocopherol, Beta-Carotene Cancer Prevention (ATBC) cohort constitutes a relatively homogeneous population of Finnish male smokers of European ancestry, which may limit the generalizability of our findings to other populations. Residual confounding by unidentified metabolic or other factors such as chronic health conditions is possible, although we extensively adjusted our models for several potential confounders including diabetes, body mass index (BMI), serum cholesterol, smoking intensity, and alcohol consumption, and the findings were unchanged after adjusting for serum creatinine, an important indicator of kidney function.

Our study identified a large number of metabolites in various metabolic pathways associated with serum retinol concentration, some of which directly relate to prostate cancer risk associations and other known biological functions. Lipid and amino acid biochemical classes are heavily represented, which may provide some molecular insights into the role of retinol in human health and disease. In particular, lipid bioactivity related to inositol, lysolipids and sterol/steroid compounds, may potentially provide biological clues relevant to the positive association between circulating retinol and the development of prostate cancer. Further studies in diverse populations are warranted to re-examine these findings.

## Materials and methods

### Study population

The ATBC Study has been described in detail elsewhere^73^. In brief, it is a 2 × 2 factorial randomized controlled trial in which 29,133 male Finish smokers aged 50–69 were assigned to receive either alpha-tocopherol (dl-α-tocopheryl-acetate, 50 mg/day), beta-carotene (20 mg/day), both vitamins, or placebo. Participants received the study capsules for a median of 6.1 years (range 5–8 years) until the trial ended on 30 April 1993. Information on general risk factors, smoking, and medical history was obtained through a questionnaire at study entry along with a validated food-frequency questionnaire. Serum was collected at baseline (i.e., pre-supplementation and years prior to cancer diagnoses) after overnight fast was protected from light and stored at −70 °C until assayed. Incident cancer diagnosed during 20 years of follow-up were identified from the Finnish Cancer Registry. All participants provided written informed consent. The trial was approved by institutional review boards at the U.S. National Cancer Institute and the Finnish National Public Health Institute. All methods were performed in accordance with the relevant guidelines and regulations.

The present analysis is based on participants included in seven on-going or completed analyses (case-control and other, subsequently referred to as “metabolomic sets”) nested within the ATBC Study^66, 74, 75^. These included cases of prostate, lung, esophagus, and stomach cancer, along with their controls, as well as controls from a pancreatic cancer study. After excluding duplicate participants, 1,282 were included in the present analysis.

### Laboratory analysis

Baseline serum retinol concentration was measured using an isocratic high-performance liquid chromatography platform^76^. Assays were carried out from 1986–88 in a dedicated laboratory at the National Public Health Institute in Helsinki, Finland, which was certified by a National Institute of Standards and Technology quality-control testing program. Serum samples were subsequently analyzed at Metabolon, Inc. (Durham, N.C.) using ultrahigh performance liquid chromatography (LC)/mass spectroscopy (MS) and gas chromatography (GC)/mass spectrometry (MS) as described before^66, 74, 75^. The methods of sample accessioning, sample preparation, quality control, data extraction and compound identification have also been described in detail^77^. Briefly, each of the samples were analyzed using three different analytical conditions: ultrahigh performance LC-MS/MS ( + electrospray ionization (ESI)), ultrahigh performance LC-MS/MS (-ESI), and GC-MS. To remove proteins, dissociate small molecules bound to protein or trapped in the precipitated protein matrix, and to recover chemically diverse metabolites, each of the samples were extracted using an aqueous methanol extraction procedure. The methanol contained four recovery standards (DL-2-fluorophenylglycine, tridecanoic acid, d6-cholesterol and 4-chlorophenylalanine) to allow confirmation of extraction efficiency. For each sample, four aliquots were obtained from the extract and dried. Two aliquots of each sample were then reconstituted in 50 µl of 6.5 mM ammonium bicarbonate in water (pH 8) for the negative ion analysis, and the other two aliquots of each were reconstituted using 50 µl 0.1% formic acid in water (pH ~3.5) for the positive ion analysis. The resulting extracts were divided into fractions for analysis by ultrahigh performance LC/MS/MS (positive mode), ultrahigh performance LC/MS/MS (negative mode), and GC/MS. Samples were placed briefly on a TurboVap® (Zymark) to remove the organic solvent. Each sample was then frozen and dried under vacuum. The samples were then prepared for the appropriate instrument, either ultrahigh performance LC/MS/MS or GC/MS. The raw data was further extracted, peak-identified and quality control (QC) processed using Metabolon’s hardware and software. Internal controls included extraction process (5 recovery standards), injection (up to 11 standards), and alignment standards for quality assurance/quality control procedures to control for experimental variability. Compounds were identified by comparison to library entries of purified standards or recurrent unknown entities. Metabolon maintains a library based on authenticated standards that contains the retention time/index (RI), mass to charge ratio (m/z), and chromatographic data (including MS/MS spectral data) on all molecules present in the library. Furthermore, biochemical identifications are based on three criteria: retention index within a narrow RI window of the proposed identification, accurate mass match to the library + /− 0.005 amu, and the MS/MS forward and reverse scores between the experimental data and authentic standards. The MS/MS scores are based on a comparison of the ions present in the experimental spectrum to the ions present in the library spectrum. More than 2400 commercially available purified standard compounds have been acquired and used in both the LC and GC platforms for determination of their analytical characteristics^78^.

Batch variability was standardized by dividing the signal strength by the batch median value for each metabolite and subject. Metabolite signal strength were log-transformed and centered according to normalization. Within each metabolomic set, metabolite values below the limit of detection were imputed to have the minimum of all non-missing values.

After excluding metabolites that had fewer than 10 non-missing values across all metabolomic sets, or single-value metabolites in individual metabolomic set, 947 identified compounds remained eligible for analysis. Based upon a standard chemical classification scheme, 920 of the metabolites were categorized in eight mutually exclusive chemical classes: amino acids and amino acid derivatives (referred to as “amino acids”), carbohydrates, cofactors and vitamins, energy metabolites, lipids, nucleotides, peptides, and xenobiotics. Blinded quality control samples (9%) from a pooled sample were included in each batch to assess the technical reliability of data, and coefficient of variation (CV) was calculated. The median and interquartile range of the CV% across the metabolites was 9% (4%-20%). These CVs are similar to those previously observed for blood samples analyzed by the same laboratory^77, 78^, and previous studies have reported a high reliability for the metabolomics platform used in the present study^79^.

### Statistical analysis

We investigated the association between serum levels of retinol and each metabolite, using linear regression adjusted for age at randomization (continuous), BMI (continuous), case status (binary, case or control status in each metabolomic set), metabolomic sets (categorical), serum cholesterol (continuous), number of cigarettes per days (continuous), and baseline alcohol consumption (continuous). We further performed additional analysis to adjust for creatinine in the model. For each model, we computed the standardized beta-coefficient which represents the change in the level of each metabolite per 1-SD increase in the levels of retinol.

For all analyses, we used log-transformed metabolite concentrations. To control for multiple comparisons (n = 947 retinol-metabolite associations), we applied the Bonferroni correction^80^ using *p* = 0.05/947 = 5.3 × 10^−5^ as the adjusted significance threshold. We further performed sensitivity analyses additionally adjusted for storage time (from blood collection to metabolomics measurement).

Within each metabolomic set, we applied Gene-Set Analysis (GSA), which is a pathway analysis method, to examine whether pre-defined metabolic pathways including super- and sub-pathways were related to retinol within individual metabolomic sets^81^. For Z values (i.e. Z_1_ to Z_s_) tested from S metabolites in a pre-defined pathway, GSA examines the “maxmean” statistic max that is the average of all Z values (both positive and negative) and calculates the *p* values by 10,000 permutations. With the seven individual metabolomic sets, we used Fisher’s method, namely sum of logs method, to combine *p* value for each pre-defined pathway.

Analyses were performed in SAS 9.4, and R 3.2.3. All statistical tests and reported *p* values are two-sided.

## Electronic supplementary material

Supplementary Information

## References

[1] Sommer A, Vyas KS (2012). A global clinical view on vitamin A and carotenoids. Am J Clin Nutr.

[2] Bates CJ (1995). Vitamin A. Lancet.

[3] Sommer A, W. K. Vitamin A deficiency: health, survival, and vision. New York: Oxford University Press. (1996.).

[4] D’Ambrosio DN, Clugston RD, Blaner WS (2011). Vitamin A metabolism: an update. Nutrients.

[5] Sporn MB, R. A., Goodman DS, editors. The retinoids: biology, chemistry, and medicine. New York: Raven Press. (1994).

[6] Dadon Bar-El, S. & Reifen, R. Vitamin A and the Epigenome. *Critical Reviews in Food Science and Nutrition*, 00–00, 10.1080/10408398.2015.1060940 (2015). 10.1080/10408398.2015.1060940 26565606

[7] Underwood BA (2004). Vitamin A deficiency disorders: international efforts to control a preventable “pox”. J Nutr.

[8] Sommer A (2008). Vitamin a deficiency and clinical disease: an historical overview. J Nutr.

[9] Willis MS, Wians FH (2003). The role of nutrition in preventing prostate cancer: a review of the proposed mechanism of action of various dietary substances. Clin Chim Acta.

[10] Vitamin A (1980). retinol, carotene, and cancer prevention. Br Med J.

[11] Hunter DJ (1993). A prospective study of the intake of vitamins C, E, and A and the risk of breast cancer. N Engl J Med.

[12] Krinsky NI, Johnson EJ (2005). Carotenoid actions and their relation to health and disease. Mol Aspects Med.

[13] Peehl DM, Feldman D (2003). The role of vitamin D and retinoids in controlling prostate cancer progression. Endocr Relat Cancer.

[14] Guallar E, Stranges S, Mulrow C, Appel LJ, Miller ER (2013). Enough is enough: Stop wasting money on vitamin and mineral supplements. Ann Intern Med.

[15] Taylor PR, Greenwald P (2005). Nutritional interventions in cancer prevention. J Clin Oncol.

[16] Steinmetz KA, Potter JD (1991). Vegetables, fruit, and cancer. I. Epidemiology. Cancer Causes Control.

[17] Gaziano JM (1995). A prospective study of consumption of carotenoids in fruits and vegetables and decreased cardiovascular mortality in the elderly. Ann Epidemiol.

[18] Manson JE, Gaziano JM, Jonas MA, Hennekens CH (1993). Antioxidants and cardiovascular disease: a review. J Am Coll Nutr.

[19] Brazionis L, Walker KZ, Itsiopoulos C, O’Dea K (2012). Plasma retinol: a novel marker for cardiovascular disease mortality in Australian adults. Nutr Metab Cardiovasc Dis.

[20] Fortmann SP, Burda BU, Senger CA, Lin JS, Whitlock EP (2013). Vitamin and mineral supplements in the primary prevention of cardiovascular disease and cancer: An updated systematic evidence review for the U.S. Preventive Services Task Force. Ann Intern Med.

[21] Ioannidis JP (2005). Contradicted and initially stronger effects in highly cited clinical research. JAMA.

[22] Mondul AM (2011). Serum retinol and risk of prostate cancer. Am J Epidemiol.

[23] Key TJ (2015). Carotenoids, retinol, tocopherols, and prostate cancer risk: pooled analysis of 15 studies. Am J Clin Nutr.

[24] Omenn GS (1996). Effects of a combination of beta carotene and vitamin A on lung cancer and cardiovascular disease. N Engl J Med.

[25] Sesso HD, Buring JE, Norkus EP, Gaziano JM (2005). Plasma lycopene, other carotenoids, and retinol and the risk of cardiovascular disease in men. Am J Clin Nutr.

[26] Albanes D (1996). Alpha-Tocopherol and beta-carotene supplements and lung cancer incidence in the alpha-tocopherol, beta-carotene cancer prevention study: effects of base-line characteristics and study compliance. J Natl Cancer Inst.

[27] U.S. Preventive Services Task Force. Internet: http://www.uspreventiveservicestaskforce.org/Page/Document/RecommendationStatementFinal/vitamin-supplementation-to-prevent-cancer-and-cvd-counseling.

[28] Wright ME (2010). Effects of beta-carotene supplementation on molecular markers of lung carcinogenesis in male smokers. Cancer Prev Res (Phila).

[29] Liu C, Russell RM, Wang XD (2003). Exposing ferrets to cigarette smoke and a pharmacological dose of beta-carotene supplementation enhance *in vitro* retinoic acid catabolism in lungs via induction of cytochrome P450 enzymes. J Nutr.

[30] Goodman GE, Alberts DS, Meyskens FL (2008). Retinol, vitamins, and cancer prevention: 25 years of learning and relearning. J Clin Oncol.

[31] Serum retinol concentrations for determining the prevalence of vitamin A deficiency in populations. Vitamin and mineral nutrition information system. *Genva*, *Switzerland: World Health Organization* (2011).

[32] Underwood BA (1994). Hypovitaminosis A: international programmatic issues. J Nutr.

[33] Fang L, Parti R, Hu P (2011). Characterization of N-acetyltryptophan degradation products in concentrated human serum albumin solutions and development of an automated high performance liquid chromatography-mass spectrometry method for their quantitation. J Chromatogr A.

[34] Wang Q, Liu D, Song P, Zou MH (2015). Tryptophan-kynurenine pathway is dysregulated in inflammation, and immune activation. Front Biosci (Landmark Ed).

[35] Santhanam S, Alvarado DM, Ciorba MA (2016). Therapeutic targeting of inflammation and tryptophan metabolism in colon and gastrointestinal cancer. Transl Res.

[36] Aksnes H, Hole K, Arnesen T (2015). Molecular, cellular, and physiological significance of N-terminal acetylation. Int Rev Cell Mol Biol.

[37] Eberharter A, Becker PB (2002). Histone acetylation: a switch between repressive and permissive chromatin. Second in review series on chromatin dynamics. EMBO Rep.

[38] Verdone L, Caserta M, Di Mauro E (2005). Role of histone acetylation in the control of gene expression. Biochem Cell Biol.

[39] Berry DC, Soltanian H, Noy N (2010). Repression of cellular retinoic acid-binding protein II during adipocyte differentiation. J Biol Chem.

[40] Wang YX (2003). Peroxisome-proliferator-activated receptor delta activates fat metabolism to prevent obesity. Cell.

[41] Schwarz EJ, Reginato MJ, Shao D, Krakow SL, Lazar MA (1997). Retinoic acid blocks adipogenesis by inhibiting C/EBPbeta-mediated transcription. Mol Cell Biol.

[42] Neele DM, de Wit EC, Princen HM (1999). Inhibition of apolipoprotein(a) synthesis in cynomolgus monkey hepatocytes by retinoids via involvement of the retinoic acid receptor. Biochem Pharmacol.

[43] Berry DC, Noy N (2012). Signaling by vitamin A and retinol-binding protein in regulation of insulin responses and lipid homeostasis. Biochim Biophys Acta.

[44] Toker A, Cantley LC (1997). Signalling through the lipid products of phosphoinositide-3-OH kinase. Nature.

[45] Laserna EJ (2009). Proteomic analysis of phosphorylated nuclear proteins underscores novel roles for rapid actions of retinoic acid in the regulation of mRNA splicing and translation. Mol Endocrinol.

[46] Krapels IP (2004). Myo-inositol, glucose and zinc status as risk factors for non-syndromic cleft lip with or without cleft palate in offspring: a case-control study. BJOG.

[47] Tsui MM, York JD (2010). Roles of inositol phosphates and inositol pyrophosphates in development, cell signaling and nuclear processes. Adv Enzyme Regul.

[48] Liscovitch M, Cantley LC (1994). Lipid second messengers. Cell.

[49] Moolenaar WH (1999). Bioactive lysophospholipids and their G protein-coupled receptors. Exp Cell Res.

[50] Xu Y, Xiao YJ, Baudhuin LM, Schwartz BM (2001). The role and clinical applications of bioactive lysolipids in ovarian cancer. J Soc Gynecol Investig.

[51] Goetzl EJ, Dolezalova H, Kong Y, Zeng L (1999). Dual mechanisms for lysophospholipid induction of proliferation of human breast carcinoma cells. Cancer Res.

[52] Manning, T. J., Jr., Parker, J. C. & Sontheimer, H. Role of lysophosphatidic acid and rho in glioma cell motility. *Cell Motil Cytoskeleton* **45**, 185–199, doi:10.1002/(SICI)1097-0169(200003)45:3<185::AID-CM2>3.0.CO;2-G (2000). 10.1002/(SICI)1097-0169(200003)45:3<185::AID-CM2>3.0.CO;2-G 10706774

[53] Mukai M (1999). Inhibition of tumor invasion and metastasis by a novel lysophosphatidic acid (cyclic LPA). Int J Cancer.

[54] Mills GB, Moolenaar WH (2003). The emerging role of lysophosphatidic acid in cancer. Nat Rev Cancer.

[55] Liu S (2009). Expression of autotaxin and lysophosphatidic acid receptors increases mammary tumorigenesis, invasion, and metastases. Cancer Cell.

[56] Mizokami A (2004). The adrenal androgen androstenediol is present in prostate cancer tissue after androgen deprivation therapy and activates mutated androgen receptor. Cancer Res.

[57] Zhang T (2013). Identification of potential biomarkers for ovarian cancer by urinary metabolomic profiling. J Proteome Res.

[58] Asatoor AM, Armstrong MD (1967). 3-methylhistidine, a component of actin. Biochem Biophys Res Commun.

[59] Young VR, Alexis SD, Baliga BS, Munro HN, Muecke W (1972). Metabolism of administered 3-methylhistidine. Lack of muscle transfer ribonucleic acid charging and quantitative excretion as 3-methylhistidine and its N-acetyl derivative. J Biol Chem.

[60] Haverberg LN, Munro HN, Young VR (1974). Isolation and quantitation of Ntau-methylhistidine in actin and myosin of rat skeletal muscle: use of pyridine elution of protein hydrolysates on ion-exchange resins. Biochim Biophys Acta.

[61] Hillgartner FB, Morin D, Hansen RJ (1982). Effect of excessive vitamin A intake on muscle protein turnover in the rat. Biochem J.

[62] Kumar A (2010). Metabolomic analysis of serum by (1) H NMR spectroscopy in amyotrophic lateral sclerosis. Clin Chim Acta.

[63] Nicholson JK, Lindon JC, Holmes E (1999). ‘Metabonomics’: understanding the metabolic responses of living systems to pathophysiological stimuli via multivariate statistical analysis of biological NMR spectroscopic data. Xenobiotica.

[64] Sui W (2012). A proton nuclear magnetic resonance-based metabonomics study of metabolic profiling in immunoglobulin a nephropathy. Clinics (Sao Paulo).

[65] Watanabe M (2008). Consequences of low plasma histidine in chronic kidney disease patients: associations with inflammation, oxidative stress, and mortality. Am J Clin Nutr.

[66] Mondul AM (2015). Metabolomic analysis of prostate cancer risk in a prospective cohort: The alpha-tocolpherol, beta-carotene cancer prevention (ATBC) study. Int J Cancer.

[67] Huang, J. *et al*. Prospective Serum Metabolomic Profile of Prostate Cancer by Size and Extent of Primary Tumor. *Oncotarget* 8, 45190-45199, doi:10.18632/oncotarget.16775 (2017). 10.18632/oncotarget.16775 PMC5542177 28423352

[68] Huang J (2016). Serum metabolomic profiling of prostate cancer risk in the prostate, lung, colorectal, and ovarian cancer screening trial. Br J Cancer.

[69] Bergen HR (2003). Discovery of ovarian cancer biomarkers in serum using NanoLC electrospray ionization TOF and FT-ICR mass spectrometry. Dis Markers.

[70] Orvisky E (2006). Enrichment of low molecular weight fraction of serum for MS analysis of peptides associated with hepatocellular carcinoma. Proteomics.

[71] Abbasciano V (1995). Usefulness of coagulation markers in staging of gastric cancer. Cancer Detect Prev.

[72] Villanueva J (2006). Serum peptidome patterns that distinguish metastatic thyroid carcinoma from cancer-free controls are unbiased by gender and age. Mol Cell Proteomics.

[73] The ATBC Cancer Prevention Study Group (1994). The alpha-tocopherol, beta-carotene lung cancer prevention study: design, methods, participant characteristics, and compliance. Ann Epidemiol.

[74] Mondul AM (2013). Metabolomic profile of response to supplementation with beta-carotene in the Alpha-Tocopherol, Beta-Carotene Cancer Prevention Study. Am J Clin Nutr.

[75] Mondul AM (2014). 1-stearoylglycerol is associated with risk of prostate cancer: results from serum metabolomic profiling. Metabolomics.

[76] Milne DB, Botnen J (1986). Retinol, alpha-tocopherol, lycopene, and alpha- and beta-carotene simultaneously determined in plasma by isocratic liquid chromatography. Clin Chem.

[77] Evans AM, DeHaven CD, Barrett T, Mitchell M, Milgram E (2009). Integrated, nontargeted ultrahigh performance liquid chromatography/electrospray ionization tandem mass spectrometry platform for the identification and relative quantification of the small-molecule complement of biological systems. Anal Chem.

[78] Dehaven CD, Evans AM, Dai H, Lawton KA (2010). Organization of GC/MS and LC/MS metabolomics data into chemical libraries. J Cheminform.

[79] Sampson JN (2013). Metabolomics in epidemiology: sources of variability in metabolite measurements and implications. Cancer Epidemiol Biomarkers Prev.

[80] Bland JM, Altman DG (1995). Multiple significance tests: the Bonferroni method. BMJ.

[81] Subramanian A (2005). Gene set enrichment analysis: a knowledge-based approach for interpreting genome-wide expression profiles. Proc Natl Acad Sci USA.

