# Supplementary material for: Metabolomic Profiling of Serum Retinol in the Alpha-Tocopherol, Beta-Carotene Cancer Prevention (ATBC) Study
Source: Sci Rep. 2017 Sep 6;7:10601. doi: 10.1038/s41598-017-09698-w (PMC5587770; doi:10.1038/s41598-017-09698-w)
Supplement: Supplementary file 1 — Supplementary Information [file 41598_2017_9698_MOESM1_ESM.doc]

**Metabolomic Profiling of Serum Retinol in the Alpha-Tocopherol, Beta-Carotene Cancer Prevention (ATBC) Study**

**Running title: Metabolomics Profiling of Serum Retinol in ATBC Study**

Jiaqi Huang^1^*, Orestis A. Panagiotou^1^*, Gabriella M. Anic^1^, Alison M. Mondul^2^, Linda M. Liao^1^, Andriy Derkach^1^, Rachael Stolzenberg-Solomon^1^, Stephanie J. Weinstein^1^, Demetrius Albanes^1†^

1. Division of Cancer Epidemiology and Genetics, National Cancer Institute, National Institutes of Health, Bethesda, MD
2. Department of Epidemiology, University of Michigan School of Public Health, Ann Arbor, MI

* These authors contributed equally to this work.

**^†^Corresponding author:** Demetrius Albanes, Division of Cancer Epidemiology and Genetics, National Cancer Institute, 9609 Medical Center Drive-6E342, Bethesda, MD 20892, USA;

Tel: (240)276-7212; E-mail: [DAA@NIH.GOV](mailto:DAA@NIH.GOV)

**Supplementary material**

Supplemental Figure 1: Participant Flow Chart for Metabolomic Profiling Study of Serum Retinol in the Alpha-Tocopherol, Beta-Carotene Cancer Prevention (ATBC) Study.

Supplemental Figure 2: Manhattan plot of metabolite associations with serum retinol, and gene set analysis for chemical class of metabolites associations with serum retinol.

Supplemental Figure 3: Manhattan plot of gene set analysis for chemical sub-class of metabolites and associations with serum retinol.

Supplemental Table 1: Metabolites associated with serum retinol concentration at p<0.05

**Supplemental Figure 1.** Participant Flow Chart for Metabolomic Profiling Study of Serum Retinol in the Alpha-Tocopherol, Beta-Carotene Cancer Prevention (ATBC) Study.*

ATBC Study cohort: male Finnish smokers, ages 50-69

Baseline/pre-randomization fasting serum samples collected (n=29,133)

Serum retinol concentration measured on isocratic high-performance liquid chromatography platform (n=29,104)

Serum metabolomics profiling from ultra-high performance LC-MS/GC-MS platform with metabolite standardization/normalization within each batch and log-transformed

Prostate cancer nested case-control study:

200 cases

198 controls

(PMID: 25904191)

n=398

Pancreatic /Lung cancer nested case-control study:

56 cases

151 controls

(Unpublished)

n=207

Metabolomic profile study of the ATBC trial supplements:

186 control subjects

(PMID: 23803886)

n=186

Esophageal/ gastric cancer

nested case-control study:

77 cases

67 controls

(Unpublished)

n=144

Pancreatic cancer nested case-control study:

121 controls

(Unpublished)

n=121

Pancreatic cancer nested case-control study:

121 controls

(Unpublished)

n=121

Prostate cancer nested case-control study:

54 cases

51 controls

(PMID: 25254003)

n=105

Analytical study set (n=1,282)

* Sample sizes may differ from the original study sets because of some duplicate participants across the studies. The two sets of pancreatic study controls are different individuals. The metabolomic profile sets were measured (from left to right) in these years: 2014, 2014, 2012, 2013, 2011, 2012 and 2012.

**Supplemental Figure 2:** Manhattan plot of metabolite associations with serum retinol, and gene set analysis for chemical class of metabolites associations with serum retinol.


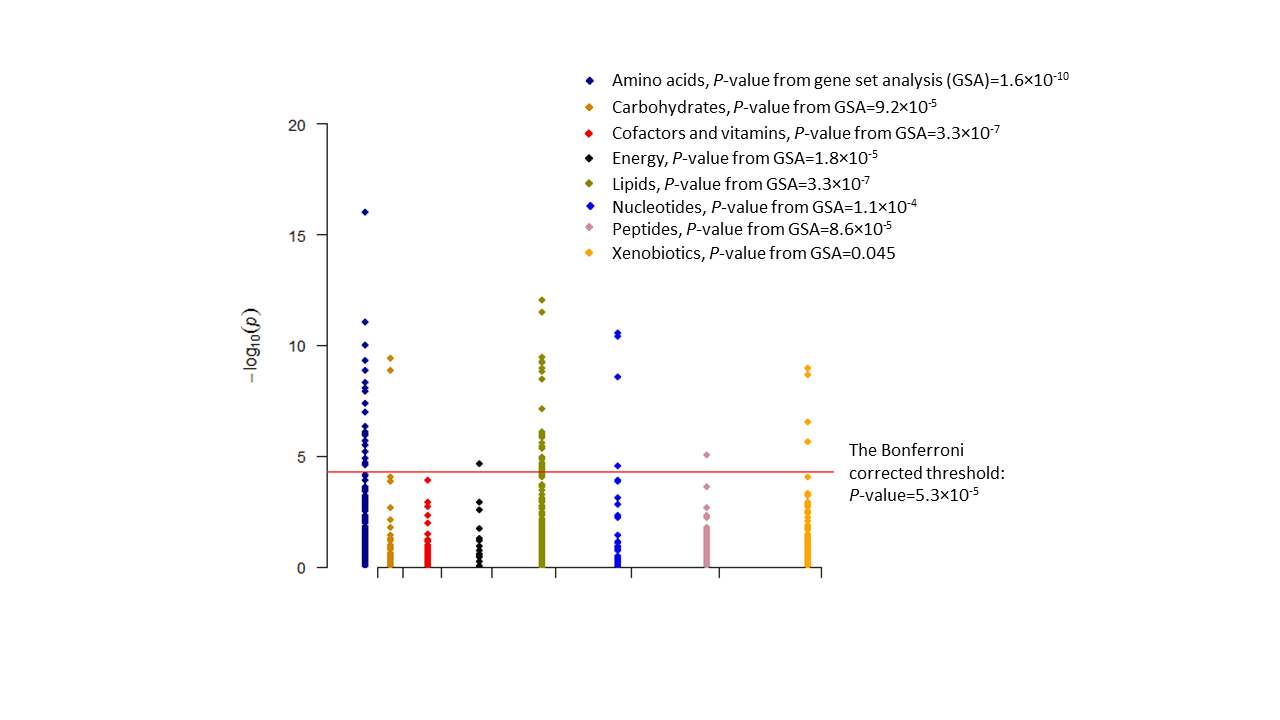


**Supplemental Figure 3:** Manhattan plot of gene set analysis for chemical sub-class of metabolites and associations with serum retinol.


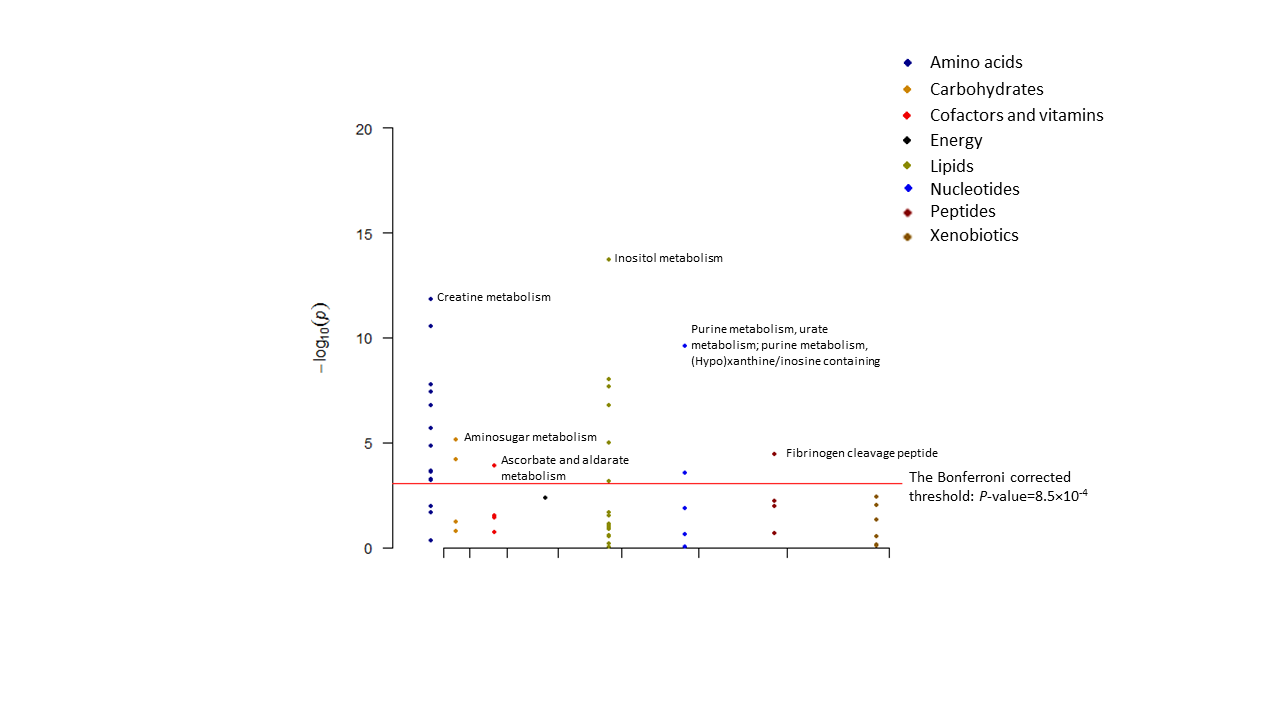


**Supplemental Table 1. Metabolites associated with serum retinol concentration at *p***<0.05

| **Metabolite** | **Estimate^1^** | **Standard error** | ***p* value** | **Chemical Class** | **Chemical Sub-Class** |
| --- | --- | --- | --- | --- | --- |
| N-Acetyltryptophan | 0.272 | 0.0321 | 9.89×10^-17^ | Amino acid | Tryptophan metabolism |
| *Myo*-inositol | 0.228 | 0.0316 | 9.82×10^-13^ | Lipid | Inositol metabolism |
| 1-Palmitoylglycerophosphoethanolamine | 0.223 | 0.0316 | 3.16×10^-12^ | Lipid | Lysolipid |
| 4-Acetamidobutanoate | 0.216 | 0.0314 | 8.87×10^-12^ | Amino acid | Guanidino and acetamido metabolism; Polyamine metabolism |
| N6-Carbamoylthreonyladenosine | 0.212 | 0.0315 | 2.91×10^-11^ | Nucleotide | Purine metabolism, guanine containing; Purine metabolism, adenine containing |
| Urate | 0.205 | 0.0308 | 4.14×10^-11^ | Nucleotide | Purine metabolism, urate metabolism; purine metabolism, (Hypo)xanthine/inosine containing |
| N-Acetyl-3-methylhistidine | 0.217 | 0.0331 | 9.84×10^-11^ | Amino acid | Histidine metabolism |
| 4-Androsten-3beta,17beta-diol monosulfate | 0.254 | 0.0397 | 3.39×10^-10^ | Lipid | Sterol/steroid |
| Erythronate | 0.198 | 0.0313 | 4.01×10^-10^ | Carbohydrate | Aminosugar metabolism |
| Homocitrulline | 0.201 | 0.0320 | 4.87×10^-10^ | Amino acid | Urea cycle; arginine and proline metabolism |
| 1-Palmitoleoylglycerophosphocholine | 0.195 | 0.0312 | 5.74×10^-10^ | Lipid | Lysolipid |
| Propionylcarnitine | 0.198 | 0.0316 | 6.20×10^-10^ | Lipid | Fatty acid metabolism (also BCAA metabolism) |
| 2-Palmitoleoylglycerophosphocholine | 0.199 | 0.0323 | 1.10×10^-9^ | Lipid | Lysolipid |
| Erythritol | 0.192 | 0.0313 | 1.10×10^-9^ | Xenobiotics | Sugar, sugar substitute, starch; food component/plant |
| N-Acetylvaline | 0.192 | 0.0314 | 1.32×10^-9^ | Amino acid | Valine, leucine and isoleucine metabolism |
| Arabitol | 0.188 | 0.0308 | 1.39×10^-9^ | Carbohydrate | Pentose metabolism |
| 5Alpha-androstan-3beta,17beta-diol disulfate | 0.189 | 0.0311 | 1.6×10^-9^ | Lipid | Sterol/steroid |
| O-sulfo-L-tyrosine | 0.217 | 0.0358 | 2.13×10^-9^ | Xenobiotics | Chemical |
| Pseudouridine | 0.191 | 0.0318 | 2.81×10^-9^ | Nucleotide | Pyrimidine metabolism, uracil containing |
| 4-Androsten-3beta,17beta-diol disulfate | 0.180 | 0.0301 | 3.35×10^-9^ | Lipid | Sterol/steroid |
| N-Acetyl-1-methylhistidine | 0.200 | 0.0339 | 4.65×10^-9^ | Amino acid | Histidine metabolism |
| 1-Methylhistidine | 0.187 | 0.0322 | 8.41×10^-9^ | Amino acid | Histidine metabolism |
| 2-Hydroxyisobutyrate | 0.205 | 0.0357 | 1.22×10^-8^ | Amino acid; Xenobiotics | Valine, leucine and isoleucine metabolism; Chemical |
| N6-Acetyllysine | 0.179 | 0.0323 | 4.23×10^-8^ | Amino acid | Lysine metabolism |
| Stearoyl-arachidonoyl-glycerophosphoethanolamine | 0.223 | 0.0409 | 7.74×10^-8^ | Lipid | Lysolipid |
| Creatinine | 0.172 | 0.0322 | 1.11×10^-7^ | Amino acid | Creatine metabolism |
| Ethyl glucuronide | 0.164 | 0.0319 | 3.00×10^-7^ | Xenobiotics | Detoxification and metabolism; chemical |
| N-Acetylthreonine | 0.162 | 0.0319 | 4.51×10^-7^ | Amino acid | Glycine, serine and threonine metabolism |
| Etiocholanolone glucuronide | 0.210 | 0.0420 | 7.91×10^-7^ | Lipid | Sterol/steroid |
| Beta-hydroxyisovaleroylcarnitine | 0.181 | 0.0363 | 8.24×10^-7^ | Amino acid | Valine, leucine and isoleucine metabolism |
| Indolelactate | 0.159 | 0.0321 | 8.99×10^-7^ | Amino acid | Tryptophan metabolism |
| 1-Linolenoylglycerophosphocholine (18:3n3) | 0.175 | 0.0354 | 9.65×10^-7^ | Lipid | Lysolipid |
| 4-Androsten-3beta,17beta-diol disulfate 2 | 0.157 | 0.0318 | 9.83×10^-7^ | Lipid | Sterol/steroid |
| Inositol 1-phosphate (I1P) | 0.148 | 0.0302 | 1.06×10^-6^ | Lipid | Inositol metabolism |
| N-Delta-acetylornithine | 0.157 | 0.0321 | 1.14×10^-6^ | Amino acid | Urea cycle; arginine and proline metabolism |
| Palmitoyl-oleoyl-glycerophosphocholine | 0.201 | 0.0412 | 1.31×10^-6^ | Lipid | Lysolipid |
| Cortisol | 0.157 | 0.0324 | 1.43×10^-6^ | Lipid | Sterol/steroid |
| N2,N5-diacetylornithine | 0.204 | 0.0425 | 2.06×10^-6^ | Amino acid | Urea cycle; arginine and proline metabolism |
| 3-Hydroxycotinine glucuronide | 0.143 | 0.0302 | 2.34×10^-6^ | Xenobiotics | Tobacco metabolite |
| 1-Oleoylglycerol (1-monoolein) | 0.160 | 0.0338 | 2.51×10^-6^ | Lipid | Monoacylglycerol |
| Urea | 0.151 | 0.0323 | 3.47×10^-6^ | Amino acid | Urea cycle; arginine and proline metabolism |
| Acetylcarnitine | 0.147 | 0.0315 | 3.73×10^-6^ | Lipid | Carnitine metabolism |
| 2-Palmitoylglycerophosphocholine | 0.148 | 0.0321 | 4.26×10^-6^ | Lipid | Lysolipid |
| Malonylcarnitine | 0.196 | 0.0424 | 4.57×10^-6^ | Lipid | Fatty acid synthesis |
| 3-Methoxytyramine sulfate | 0.197 | 0.0432 | 6.58×10^-6^ | Amino acid | Phenylalanine & tyrosine metabolism |
| Adsgegdfxaegggvr | -0.157 | 0.0353 | 9.38×10^-6^ | Peptide | Fibrinogen cleavage peptide |
| Carnitine | 0.140 | 0.0318 | 1.2×10^-5^ | Lipid | Carnitine metabolism |
| Scyllo-inositol | 0.262 | 0.0591 | 1.31×10^-5^ | Lipid | Inositol metabolism |
| Guanidinosuccinate | 0.182 | 0.0414 | 1.32×10^-5^ | Amino acid | Guanidino and acetamido metabolism |
| 1-Oleoylglycerophosphocholine | 0.137 | 0.0315 | 1.47×10^-5^ | Lipid | Lysolipid |
| 2-Myristoylglycerol  (2-monomyristin) | 0.156 | 0.0363 | 1.94×10^-5^ | Lipid | Monoacylglycerol |
| 3-Methylhistidine | 0.140 | 0.0327 | 1.97×10^-5^ | Amino acid | Histidine metabolism |
| 1-Oleoylglycerophosphoethanolamine | 0.138 | 0.0322 | 2.13×10^-5^ | Lipid | Lysolipid |
| 1-Palmitoylglycerophosphocholine | 0.138 | 0.0322 | 2.17×10^-5^ | Lipid | Lysolipid |
| 2-Palmitoylglycerol (2-monopalmitin) | 0.148 | 0.0347 | 2.33×10^-5^ | Lipid | Monoacylglycerol |
| Isovalerylcarnitine | 0.132 | 0.0310 | 2.35×10^-5^ | Amino acid | Valine, leucine and isoleucine metabolism |
| Succinylcarnitine | 0.134 | 0.0315 | 2.37×10^-5^ | Energy | Krebs cycle / TCA cycle |
| Tiglyl carnitine | 0.138 | 0.0326 | 2.56×10^-5^ | Amino acid | Valine, leucine and isoleucine metabolism |
| N2,N2-Dimethylguanosine | 0.134 | 0.0317 | 2.76×10^-5^ | Nucleotide | Purine metabolism, guanine containing |
| 21-Hydroxypregnenolone disulfate | 0.135 | 0.0321 | 2.94×10^-5^ | Lipid | Sterol/steroid |
| Palmitoyl-arachidonoyl-glycerophosphocholine | 0.165 | 0.0395 | 3.42×10^-5^ | Lipid | Lysolipid |
| 1-Linolenoylglycerol | 0.138 | 0.0332 | 3.72×10^-5^ | Lipid |  |
| 1-Stearoylglycerophosphoethanolamine | 0.131 | 0.0321 | 4.64×10^-5^ | Lipid | Lysolipid |
| Stearoyl-linoleoyl-glycerophosphoethanolamine | 0.169 | 0.0421 | 6.50×10^-5^ | Lipid | Lysolipid |
| 2-Methylbutyrylcarnitine (C5) | 0.153 | 0.0382 | 6.83×10^-5^ | Amino acid | Valine, leucine and isoleucine metabolism |
| N-Acetylserine | 0.129 | 0.0322 | 6.90×10^-5^ | Amino acid | Glycine, serine and threonine metabolism |
| Creatine | 0.128 | 0.0322 | 7.27×10^-5^ | Amino acid | Creatine metabolism |
| 3-Carboxy-4-methyl-5-propyl-2-furanpropanoate (CMPF) | 0.126 | 0.0318 | 7.59×10^-5^ | Lipid | Fatty acid, Dicarboxylate |
| 1-Myristoylglycerol (1-monomyristin) | 0.128 | 0.0322 | 7.97×10^-5^ | Lipid | Monoacylglycerol |
| Methionine sulfone | 0.169 | 0.0426 | 8.22×10^-5^ | Amino acid | Cysteine, methionine, SAM, taurine metabolism |
| Threitol | 0.126 | 0.0320 | 8.62×10^-5^ | Carbohydrate | Pentose metabolism |
| Xylonate | 0.130 | 0.0330 | 8.79×10^-5^ | Carbohydrate | Pentose metabolism |
| N-Acetylalliin | 0.171 | 0.0434 | 8.97×10^-5^ | Xenobiotics | Food component/Plant |
| Palmitoyl-arachidonoyl-Glycerophosphocholine | 0.163 | 0.0413 | 9.25×10^-5^ | Lipid | Lysolipid |
| 1,3-Dipalmitoylglycerol | 0.138 | 0.0352 | 9.42×10^-5^ | Lipid | Diacylglycerol |
| Pantothenate | 0.125 | 0.0325 | 0.000128 | Cofactors and vitamins | Pantothenate and COA metabolism |
| N6-Succinyladenosine | 0.166 | 0.0431 | 0.000129 | Nucleotide | Purine metabolism, adenine containing |
| N2-Acetyllysine | 0.132 | 0.0342 | 0.000132 | Amino acid | Lysine metabolism |
| N-Acetylputrescine | 0.129 | 0.0335 | 0.000133 | Amino acid | Polyamine metabolism |
| N4-Acetylcytidine | 0.167 | 0.0435 | 0.000137 | Nucleotide | Pyrimidine metabolism, cytidine containing |
| N-Acetylneuraminate | 0.133 | 0.0348 | 0.000139 | Carbohydrate | Aminosugar metabolism |
| 5Alpha-androstan-3alpha,17beta-diol disulfate | 0.123 | 0.0328 | 0.000196 | Lipid | Sterol/steroid |
| 1-Stearoylglycerophosphoglycerol | 0.122 | 0.0330 | 0.000236 | Lipid | Lysolipid |
| Isobutyrylcarnitine | 0.119 | 0.0324 | 0.000244 | Amino acid | Valine, leucine and isoleucine metabolism |
| 2-Stearoylglycerophosphoethanolamine | 0.116 | 0.0316 | 0.000249 | Lipid | Lysolipid |
| Prolylglycine | 0.131 | 0.0355 | 0.000253 | Peptide | Dipeptide |
| N-Acetylglutamate | 0.146 | 0.0400 | 0.000281 | Amino acid | Glutamate metabolism |
| N-Acetylphenylalanine | 0.122 | 0.0339 | 0.000318 | Amino acid | Phenylalanine & tyrosine metabolism |
| Beta-hydroxyisovalerate | 0.118 | 0.0328 | 0.000324 | Amino acid | Valine, leucine and isoleucine metabolism |
| 2-Oleoylglycerophosphoethanolamine | 0.176 | 0.0489 | 0.000362 | Lipid | Lysolipid |
| 3-Hydroxy-2-ethylpropionate | 0.116 | 0.0325 | 0.000368 | Amino acid | Valine, leucine and isoleucine metabolism |
| N-Acetyltaurine | 0.127 | 0.0357 | 0.000384 | Amino acid | Cysteine, methionine, SAM, taurine metabolism |
| Xanthurenate | 0.122 | 0.0344 | 0.000401 | Amino acid | Tryptophan metabolism |
| Triamterene | 0.120 | 0.0342 | 0.00048 | Xenobiotics | Drug |
| 1-Arachidonoylglycerophosphocholine | 0.112 | 0.0322 | 0.000548 | Lipid | Lysolipid |
| S-Allylcysteine | 0.150 | 0.0435 | 0.000606 | Xenobiotics | Food component/Plant |
| C-Glycosyltryptophan | 0.109 | 0.0318 | 0.000615 | Amino acid | Tryptophan metabolism |
| N-Acetylglutamine | 0.123 | 0.0360 | 0.000686 | Amino acid | Glutamate metabolism |
| Cysteine-glutathione disulfide | -0.111 | 0.0328 | 0.000731 | Amino acid | Glutathione metabolism |
| N1-Methyladenosine | 0.109 | 0.0322 | 0.000754 | Nucleotide | Purine metabolism, adenine containing |
| Pregnen-diol disulfate | 0.106 | 0.0316 | 0.000787 | Lipid | Sterol/steroid |
| 1-Docosahexaenoylglycerol | 0.143 | 0.0423 | 0.000788 | Lipid | Monoacylglycerol |
| Cortisone | 0.109 | 0.0322 | 0.00079 | Lipid | Sterol/steroid |
| N-Acetylmethionine | -0.102 | 0.0307 | 0.000865 | Amino acid | Cysteine, methionine, SAM, taurine metabolism |
| Taurocholenate sulfate | -0.109 | 0.0329 | 0.000885 | Lipid | Secondary bile acid metabolism |
| 5-Methylthioadenosine (MTA) | 0.105 | 0.0315 | 0.000907 | Amino acid | Polyamine metabolism |
| Deoxycholate | 0.142 | 0.0426 | 0.000914 | Lipid | Secondary bile acid metabolism |
| 1-Docosapentaenoylglycerophosphocholine | 0.165 | 0.0495 | 0.000927 | Lipid | Lysolipid |
| N-Acetylhistidine | 0.111 | 0.0337 | 0.001028 | Amino acid | Histidine metabolism |
| 2-Docosahexaenoylglcyerol | 0.177 | 0.0534 | 0.001044 | Lipid |  |
| 1-Palmitoylglycerol (1-monopalmitin) | 0.107 | 0.0325 | 0.001068 | Lipid | Monoacylglycerol |
| Aspartate | -0.107 | 0.0326 | 0.001098 | Amino acid | Alanine and aspartate metabolism |
| Campesterol | -0.104 | 0.0317 | 0.001118 | Lipid | Sterol/steroid |
| 1-Eicosapentaenoylglycerophosphoethanolamine | 0.216 | 0.0657 | 0.001141 | Lipid | Lysolipid |
| 4-Ethylphenylsulfate | 0.105 | 0.0325 | 0.001194 | Xenobiotics | Benzoate metabolism |
| 1-Palmitoleoylglycerophosphoethanolamine | 0.153 | 0.0471 | 0.001238 | Lipid | Lysolipid |
| Arabonate | 0.136 | 0.0421 | 0.001259 | Cofactors and vitamins | Ascorbate and aldarate metabolism |
| Alpha-ketoglutarate | 0.102 | 0.0315 | 0.001286 | Energy | Krebs cycle / TCA cycle |
| 1,2-Dipalmitoylglycerol | 0.114 | 0.0353 | 0.001294 | Lipid | Diacylglycerol |
| Indoleacetylglutamine | 0.108 | 0.0336 | 0.001361 | Amino acid | Tryptophan metabolism |
| Thymol sulfate | 0.111 | 0.0347 | 0.001453 | Xenobiotics | Food component/Plant |
| 7-Methylguanine | 0.100 | 0.0316 | 0.001632 | Nucleotide | Purine metabolism, guanine containing |
| N-Acetylleucine | 0.101 | 0.0320 | 0.00165 | Amino acid | Valine, leucine and isoleucine metabolism |
| Pregnanediol-3-glucuronide | 0.102 | 0.0324 | 0.001732 | Lipid | Sterol/steroid |
| N-Acetyl-beta-alanine | 0.103 | 0.0328 | 0.001772 | Amino acid; Nucleotide | Alanine and aspartate metabolism; Pyrimidine metabolism, uracil containing |
| Cysteine | 0.101 | 0.0324 | 0.001774 | Amino acid | Cysteine, methionine, SAM, taurine metabolism |
| 2-Palmitoylglycerophosphoethanolamine | 0.152 | 0.0484 | 0.001804 | Lipid | Lysolipid |
| Hydroxycotinine | 0.092 | 0.0294 | 0.001827 | Xenobiotics | Tobacco metabolite |
| S-(3-Hydroxypropyl)mercapturic acid (HPMA) | 0.127 | 0.0408 | 0.001927 | Xenobiotics | Chemical |
| N1-Methyl-2-pyridone-5-carboxamide | 0.101 | 0.0325 | 0.002069 | Cofactors and vitamins | Nicotinate and nicotinamide metabolism |
| N-Acetylkynurenine | 0.132 | 0.0426 | 0.002102 | Amino acid | Tryptophan metabolism |
| 1-Arachidonoylglycerophosphoethanolamine | 0.099 | 0.0321 | 0.002126 | Lipid | Lysolipid |
| Mannose | -0.098 | 0.0319 | 0.002136 | Carbohydrate | Fructose, mannose, galactose, starch, and sucrose metabolism |
| Phenylalanyltryptophan | 0.151 | 0.0492 | 0.002264 | Peptide | Dipeptide |
| Beta-sitosterol | -0.135 | 0.0444 | 0.002475 | Lipid | Sterol/steroid |
| N-acetyltyrosine | 0.095 | 0.0315 | 0.002642 | Amino acid | Phenylalanine & tyrosine metabolism |
| Phenol sulfate | 0.099 | 0.0329 | 0.002676 | Amino acid | Phenylalanine & tyrosine metabolism |
| 2-Methylcitrate | 0.162 | 0.0538 | 0.002714 | Energy | Krebs cycle / TCA cycle |
| Sulfate | 0.129 | 0.0432 | 0.002991 | Xenobiotics | Chemical |
| Imidazole lactate | 0.107 | 0.0364 | 0.003233 | Amino acid | Histidine metabolism |
| Palmitoleoyl sphingomyelin | -0.117 | 0.0397 | 0.003301 | Lipid | Sphingolipid metabolism |
| Caproate (6:0) | 0.096 | 0.0328 | 0.003397 | Lipid | Medium chain fatty acid |
| 3-Hydroxy-3-methylglutarate | 0.125 | 0.0424 | 0.003422 | Lipid | Mevalonate metabolism |
| Alliin | 0.101 | 0.0349 | 0.003889 | Xenobiotics | Food component/Plant |
| 5Alpha-androstan-3beta,17beta-diol monosulfate | 0.123 | 0.0432 | 0.004537 | Lipid | Sterol/steroid |
| Palmitoyl-palmitoyl-glycerophosphocholine | 0.120 | 0.0423 | 0.00466 | Lipid | Lysolipid |
| Kynurenate | 0.090 | 0.0316 | 0.004662 | Amino acid | Tryptophan metabolism |
| Glycylisoleucine | -0.154 | 0.0541 | 0.004674 | Peptide | Dipeptide |
| Dihydroorotate | 0.112 | 0.0394 | 0.004678 | Nucleotide | Pyrimidine metabolism, orotate containing |
| S-Adenosylhomocysteine (SAH) | 0.104 | 0.0366 | 0.004688 | Amino acid | Cysteine, methionine, SAM, taurine metabolism |
| 2-Oleoylglycerophosphocholine | 0.139 | 0.0489 | 0.004735 | Lipid | Lysolipid |
| Hexanoylcarnitine | 0.090 | 0.0318 | 0.004787 | Lipid | Carnitine metabolism |
| 3-Phenylpropionate (hydrocinnamate) | -0.092 | 0.0325 | 0.004838 | Amino acid | Phenylalanine & tyrosine metabolism |
| Threonate | 0.092 | 0.0326 | 0.004866 | Cofactors and vitamins | Ascorbate and aldarate metabolism |
| Flavin adenine dinucleotide (FAD) | 0.114 | 0.0405 | 0.005076 | Cofactors and vitamins | Riboflavin metabolism |
| Gulonic acid | 0.151 | 0.0536 | 0.005083 |  |  |
| 3-Methylglutarylcarnitine | 0.117 | 0.0424 | 0.005745 | Amino acid | Lysine metabolism |
| Phytanate | -0.160 | 0.0577 | 0.005984 | Xenobiotics | Food component/Plant |
| 9-Methyluric acid | 0.174 | 0.0629 | 0.006152 | Nucleotide | Purine metabolism, urate metabolism; purine metabolism, (Hypo)xanthine/inosine containing |
| Betaine | -0.087 | 0.0318 | 0.006153 | Amino acid | Glycine, serine and threonine metabolism |
| Adpsgegdfxaegggvr | -0.162 | 0.0592 | 0.006515 | Peptide | Fibrinogen cleavage peptide |
| Lathosterol | 0.101 | 0.0374 | 0.006897 | Lipid | Sterol/steroid |
| 1-Linoleoylglycerophosphoethanolamine | 0.087 | 0.0322 | 0.007186 | Lipid | Lysolipid |
| N-Acetylaspartate (NAA) | 0.094 | 0.0353 | 0.00773 | Amino acid | Alanine and aspartate metabolism |
| 2-Arachidonoylglycerophosphoethanolamine | 0.131 | 0.0489 | 0.00794 | Lipid | Lysolipid |
| 5Alpha-pregnan-3(alpha or beta),20beta-diol disulfate | 0.116 | 0.0435 | 0.007955 | Lipid | Sterol/steroid |
| Xylitol | 0.089 | 0.0333 | 0.007956 | Carbohydrate | Pentose metabolism |
| N-Acetylalanine | 0.086 | 0.0325 | 0.007957 | Amino acid | Alanine and aspartate metabolism |
| 1-Docosahexaenoylglycerophosphocholine | 0.132 | 0.0495 | 0.008193 | Lipid | Lysolipid |
| 1-Pentadecanoylglycerol (1-monopentadecanoin) | 0.097 | 0.0366 | 0.008197 | Lipid | Monoacylglycerol |
| 3-Methylglutaconate | 0.115 | 0.0435 | 0.0084 | Amino acid | Lysine metabolism; Valine, leucine and isoleucine metabolism |
| Metoprolol | 0.085 | 0.0325 | 0.008933 | Xenobiotics | Drug |
| 2-Hydroxystearate | -0.084 | 0.0322 | 0.009182 | Lipid | Fatty acid, Monohydroxy |
| 2-Hydroxy-3-methylvalerate | 0.084 | 0.0325 | 0.009861 | Amino acid | Valine, leucine and isoleucine metabolism |
| Picolinate | 0.179 | 0.0694 | 0.010425 | Amino acid | Tryptophan metabolism |
| Bilirubin (Z,Z) | 0.083 | 0.0323 | 0.010597 | Cofactors and vitamins | Hemoglobin and porphyrin metabolism |
| 2-Methylmalonyl carnitine | 0.088 | 0.0344 | 0.010992 | Lipid | Carnitine metabolism; Fatty acid synthesis |
| 1-Linoleoylglycerol (1-monolinolein) | 0.082 | 0.0325 | 0.011443 | Lipid | Monoacylglycerol |
| 5Alpha-androstan-3alpha,17beta-diol monosulfate | 0.109 | 0.0436 | 0.01273 | Lipid | Sterol/steroid |
| Piperine | 0.079 | 0.0318 | 0.01381 | Xenobiotics | Food component/Plant |
| Stearidonate (18:4n3) | 0.079 | 0.0321 | 0.014199 | Lipid | Long chain fatty acid; polyunsaturated fatty acid (N3 and N6) |
| Betonicine | 0.108 | 0.0441 | 0.014537 | Xenobiotics | Food component/Plant |
| 16-Hydroxypalmitate | 0.127 | 0.0517 | 0.014608 | Lipid | Fatty acid, Monohydroxy |
| 1-Eicosatrienoylglycerophosphoethanolamine | 0.169 | 0.0691 | 0.014881 | Lipid | Lysolipid |
| Salicylate | 0.080 | 0.0327 | 0.014885 | Xenobiotics | Drug |
| 1-Palmitoylglycerophosphoglycerol | 0.089 | 0.0364 | 0.015106 | Lipid | Glycerophosphodiester/ Lysolipid |
| 1Ooleoylglycerophosphoglycerol | 0.086 | 0.0354 | 0.015112 | Lipid | Glycerophosphodiester/ Lysolipid |
| Cotinine | -0.073 | 0.0300 | 0.015835 | Xenobiotics | Tobacco metabolite |
| 3-Methylglutarylcarnitine | 0.105 | 0.0433 | 0.015975 | Amino acid | Lysine metabolism |
| Vanillylmandelate (VMA) | 0.092 | 0.0381 | 0.016032 | Amino acid | Phenylalanine & tyrosine metabolism |
| Cysteine | 0.087 | 0.0362 | 0.016452 | Amino acid | Cysteine, methionine, SAM, taurine metabolism |
| Glycerate | 0.079 | 0.0327 | 0.016626 | Carbohydrate | Glycolysis, gluconeogenesis, pyruvate metabolism |
| 1-Eicosatrienoylglycerophosphocholine | 0.118 | 0.0492 | 0.01683 | Lipid | Lysolipid |
| DSGEGDFXAEGGGVR | -0.075 | 0.0312 | 0.017006 | Peptide | Fibrinogen cleavage peptide |
| Glutamate | 0.072 | 0.0303 | 0.017258 | Amino acid | Glutamate metabolism |
| 2-Aminophenol sulfate | 0.078 | 0.0328 | 0.017475 | Xenobiotics | Chemical |
| Alpha-hydroxyisovaleroyl carnitine | 0.169 | 0.0710 | 0.018091 | Amino acid | Valine, leucine and isoleucine metabolism |
| Citrate | -0.076 | 0.0323 | 0.01843 | Energy | Krebs cycle / TCA cycle |
| 3-Methylurate | 0.179 | 0.0758 | 0.019196 | Xenobiotics | Xanthine metabolism |
| 1-Stearoylglycerol (1-monostearin) | 0.075 | 0.0322 | 0.019823 | Lipid | Monoacylglycerol |
| Dopamine sulfate | 0.102 | 0.0439 | 0.019909 | Amino acid | Phenylalanine & tyrosine metabolism |
| Tyrosine | -0.072 | 0.0309 | 0.020062 | Amino acid | Phenylalanine & tyrosine metabolism |
| Leucylalanine | -0.115 | 0.0494 | 0.020552 | Peptide | Dipeptide |
| 11-Ketoetiocholanolone glucuronide | 0.098 | 0.0424 | 0.020616 | Lipid | Sterol/steroid |
| 3-(N-Acetyl-L-cystein-S-yl) acetaminophen | 0.244 | 0.1044 | 0.02109 | Xenobiotics | Drug |
| Valerylcarnitine | 0.078 | 0.0339 | 0.021198 | Lipid | Fatty acid METABOLISM; Carnitine metabolism |
| Glutamine | -0.076 | 0.0330 | 0.021523 | Amino acid | Glutamate metabolism |
| 3-Indoxyl sulfate | 0.076 | 0.0329 | 0.021544 | Amino acid | Tryptophan metabolism |
| 2-Ethylhexanoate | 0.111 | 0.0480 | 0.022115 | Xenobiotics | Chemical |
| 1-Methylimidazoleacetate | 0.080 | 0.0347 | 0.022126 | Amino acid | Histidine metabolism |
| Glycolate (hydroxyacetate) | -0.083 | 0.0364 | 0.022149 | Xenobiotics | Chemical |
| 4-Amino-2-hydroxybutyrate | 0.241 | 0.1043 | 0.022832 | Amino acid | Cysteine, methionine, SAM, taurine metabolism |
| 1-Docosahexaenoylglycerophosphoethanolamine | 0.150 | 0.0664 | 0.024933 | Lipid | Lysolipid |
| 3Beta,7alpha-dihydroxy-5-cholestenoate | -0.095 | 0.0422 | 0.024991 | Lipid | Secondary bile acid metabolism |
| HWESASXX | -0.071 | 0.0319 | 0.025126 | Peptide | Polypeptide |
| 3-Hydroxyindolin-2-one | 0.121 | 0.0540 | 0.025802 |  |  |
| 2-Aminobutyrate | 0.072 | 0.0322 | 0.025869 | Amino acid | Butanoate metabolism; Cysteine, methionine, SAM, taurine metabolism |
| Cys-gly, oxidized | 0.072 | 0.0323 | 0.02639 | Peptide; Amino acid | Dipeptide derivative; Glutathione metabolism |
| Glycerophosphorylcholine (GPC) | -0.071 | 0.0318 | 0.026709 | Lipid | Glycerolipid metabolism; Phospholipid metabolism |
| Gamma-glutamyl-2-aminobutyrate | 0.085 | 0.0383 | 0.026837 | Peptide | Gamma-glutamyl amino acid |
| Gamma-glutamylglutamine | -0.072 | 0.0325 | 0.027179 | Peptide | Gamma-glutamyl amino acid |
| Palmitoylcarnitine | 0.071 | 0.0321 | 0.027334 | Lipid | Carnitine metabolism |
| Indolepropionate | -0.072 | 0.0330 | 0.028928 | Amino acid | Tryptophan metabolism |
| pregnenolone sulfate | 0.069 | 0.0318 | 0.029421 | Lipid | Sterol/steroid |
| Alpha-hydroxyisovalerate | 0.067 | 0.0308 | 0.029976 | Amino acid | Valine, leucine and isoleucine metabolism |
| Docosapentaenoate (n3 DPA; 22:5n3) | 0.070 | 0.0320 | 0.030172 | Lipid | Essential fatty acid; polyunsaturated fatty acid (N3 and N6) |
| Hydroxyisovaleroyl carnitine | 0.158 | 0.0725 | 0.030188 | Amino acid | Valine, leucine and isoleucine metabolism |
| 5Alpha-pregnan-3beta,20alpha-diol disulfate | 0.070 | 0.0324 | 0.032027 | Lipid | Sterol/steroid |
| Stearoyl-arachidonoyl-glycerophosphoinositol | 0.090 | 0.0422 | 0.032421 | Lipid | Lysolipid |
| 2-Myristoylglycerophosphocholine | 0.105 | 0.0492 | 0.033242 | Lipid | Lysolipid |
| Diethanolamine | -0.219 | 0.1023 | 0.034829 | Lipid | Glycerolipid metabolism |
| 1-Myristoleoylglycerophosphocholine (14:1) | 0.143 | 0.0673 | 0.035114 | Lipid | Lysolipid |
| 2,3-Dihydroxyisovalerate | 0.076 | 0.0361 | 0.035234 | Cofactors and vitamins; Xenobiotics | Pantothenate and COA metabolism; Food component/Plant |
| 4-Hydroxycoumarin | -0.092 | 0.0438 | 0.03577 | Xenobiotics | Drug |
| N-Acetylcarnosine | 0.069 | 0.0330 | 0.035836 | Peptide | Dipeptide derivative; |
| Myristoleoyl sphingomyelin | -0.083 | 0.0397 | 0.035904 | Lipid | Sphingolipid metabolism |
| Gentisate | 0.073 | 0.0348 | 0.036097 | Amino acid | Phenylalanine & tyrosine metabolism |
| Indole-3-carboxylic acid | -0.091 | 0.0433 | 0.036392 | Amino acid | Tryptophan metabolism |
| 1-Palmitoylglycerophosphate | 0.095 | 0.0456 | 0.037958 | Lipid | Lysolipid |
| Adenosine 5'-monophosphate (AMP) | 0.086 | 0.0415 | 0.038399 | Nucleotide | Purine metabolism, adenine containing |
| Indolepyruvate | 0.134 | 0.0643 | 0.038831 | Amino acid | Tryptophan metabolism |
| Gamma-glutamylisoleucine | 0.064 | 0.0310 | 0.039316 | Peptide | Gamma-glutamyl amino acid |
| Docosahexaenoate (DHA; 22:6n3) | 0.066 | 0.0321 | 0.039391 | Lipid | Essential fatty acid; polyunsaturated fatty acid (N3 and N6) |
| Ribitol | 0.068 | 0.0329 | 0.039438 | Carbohydrate | Pentose metabolism |
| 1-Myristoylglycerophosphocholine | 0.100 | 0.0489 | 0.042266 | Lipid | Lysolipid |
| 1-Oleoylglycerophosphoinositol | 0.066 | 0.0323 | 0.042438 | Lipid | Lysolipid |
| 2-Linoleoylglycerol (2-monolinolein) | 0.067 | 0.0329 | 0.042599 | Lipid | Monoacylglycerol |
| P-Acetamidophenylglucuronide | 0.077 | 0.0381 | 0.043392 | Xenobiotics | Drug |
| 3-Dehydrocarnitine | -0.128 | 0.0632 | 0.043486 | Lipid | Carnitine metabolism |
| 1-Stearoylplasmenylethanolamine | -0.118 | 0.0585 | 0.044142 | Lipid | Lysolipid |
| Beta-alanine | 0.066 | 0.0330 | 0.044412 | Amino acid; Nucleotide | Alanine and aspartate metabolism; Pyrimidine metabolism, uracil containing |
| Isovalerylglycine | 0.075 | 0.0371 | 0.044698 | Amino acid | Valine, leucine and isoleucine metabolism |
| Estrone 3-sulfate | 0.076 | 0.0382 | 0.045486 | Lipid | Sterol/steroid |
| N-Acetylcitrulline | 0.088 | 0.0438 | 0.04559 | Amino acid | Urea cycle; arginine and proline metabolism |
| 17Alpha-hydroxypregnanolone glucuronide | 0.086 | 0.0431 | 0.045616 | Lipid | Sterol/steroid |
| 1-Linolenoylglycerophosphoethanolamine (18:3n3) | 0.135 | 0.0670 | 0.045864 | Lipid | Lysolipid |
| Stachydrine | 0.065 | 0.0330 | 0.047573 | Xenobiotics | Food component/Plant |
| Phenylalanylglycine | 0.065 | 0.0326 | 0.047911 | Peptide | Dipeptide |
| 7-Ketodeoxycholate | 0.080 | 0.0403 | 0.047957 | Lipid | Secondary bile acid metabolism |
| 2-Hydroxypalmitate | -0.064 | 0.0323 | 0.048065 | Lipid | Fatty acid, Monohydroxy |
| 4-Vinylphenol sulfate | 0.064 | 0.0323 | 0.048696 | Xenobiotics | Benzoate metabolism |
| Thyroxine | 0.084 | 0.0426 | 0.048856 | Amino acid | Phenylalanine & tyrosine metabolism |
| Glycodeoxycholate | 0.064 | 0.0325 | 0.049148 | Lipid | Secondary bile acid metabolism |

^1^Estimate is the standardized beta-coefficient
